# Mechanistic formation of hazardous molecular heterocyclic amines from high temperature pyrolysis of model biomass materials: cellulose and tyrosine

**DOI:** 10.1186/s13065-019-0644-1

**Published:** 2019-11-08

**Authors:** Samuel K. Kirkok, Joshua K. Kibet, Francis Okanga, Thomas Kinyanjui, Vincent Nyamori

**Affiliations:** 1 Department of Chemistry, Egerton University, Egerton, P.O Box 536, Nakuru, 20115 Kenya; 2 School of Chemistry and Physics, University of KwaZulu-Natal, Westville Campus, Private Bag X54001, Durban, 4000 South Africa

**Keywords:** Biomass materials, Heterocyclic amines, Mutagens, 1-naphthyl isocyanate

## Abstract

**Background:**

Research inventories on the co-pyrolysis of major biomass components such as cellulose with amino acid materials is scarce in literature despite the fact that such studies are critical in understanding toxic product relations from high temperature cooking, combustion of bio-fuels, cigarette smoking and forest fires. This paper explores, quantitatively, the yields of heterocyclic nitrogenated molecular reaction products of grave mutagenetic concern from the co-pyrolysis of model biomass materials; tyrosine and cellulose. Research has established that heterocyclic amines such as isocyanates are mutagens as well precursors for asthma, and other respiratory disorders.

**Methods:**

An equimassic mixture of tyrosine and cellulose (50 ± 2 mg) by weight were pyrolyzed in a tubular quartz reactor in flowing nitrogen at 1 atm. Besides, varying combinations of tyrosine and cellulose in the ratios 3:1 and 1:3 were also explored for comparison. The reaction time was set at 2 s so as to simulate combustions events in nature. The pyrolysate was collected over 5 mL dichloromethane and characterized using a gas chromatograph coupled to a mass spectrometer detector.

**Results:**

Evidently, it was noted that 1-methylindazole was released in high yields at 300 °C, constituting ~ 300 µg in the entire pyrolysis temperature range (200–700 °C). Nonetheless, isoindazole gave the highest yield ~ 730 µg while 1-naphthyl isocyanate gave a total yield of ~ 336 µg in the same temperature range. Remarkably, the change in char yield between 300 and 450 °C for the pyrolysis of 25% tyrosine in 75% cellulose was found to be ~ 48% whereas the change in char yield for the pyrolysis of 75% tyrosine in 25% cellulose was 49%.

**Conclusion:**

The char and tar yields considered important residues of biomass burning have been reported in this study and found to be consistent with other research output in literature. The striking similarities of % yield of char across all temperatures for various combinations was the most significant observation in this investigation—char yield was independent of the mixing ratio during pyrolysis. From a mechanistic standpoint, it was noted that tyrosine inhibited cellulose based nitrogenated products. Thus N-products dominated the O-products.
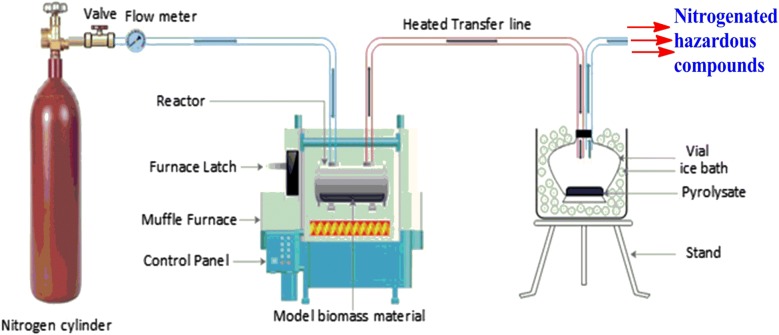

## Introduction

In the search for possible correlation between gas-phase pollutants and medical ailments such as cancer, oxidative stress and chronic bronchitis, the highly mutagenic heterocyclic amines present in cooked foods, bio-fuels and various combustion systems including municipal waste incineration, forest fires and warhead explosives have attracted significant attention in environmental toxicology and pollution research [1]. Previous studies have established that heterocyclic amines from high temperature cooking and biomass burning are hazardous to biological systems [2, 3]. On the other hand, there is significant evidence that isocyanates such as 1-naphthyl isocyanate reported in this study may cause pneumonia, asthma, and other grave respiratory diseases [4–6]. Furthermore, such studies have demonstrated that biological uptake of isocyanates may occur via dermal absorption or inhalation of airborne isocyanates [7]. Whereas it is important to note that a myriad of pyrolysis products were detected in this study only selected nitrogenated products of environmental concern will thoroughly be investigated.

**Fig. 1:**
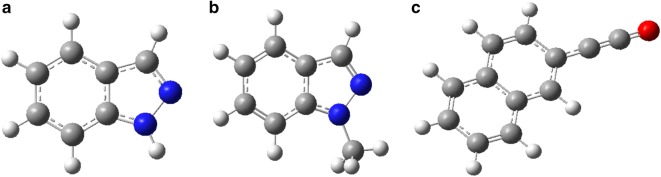
Optimized structures of **a** isoindazole, **b** 1-methyl indazole and **c** 1-naphthyl isocyanate

There exist copious data regarding the thermal degradation of cellulose in literature [8–10] but the inventory for the mechanistic degradation of tyrosine and the cross reaction mechanisms during the co-pyrolysis of cellulose and tyrosine is largely lacking primarily because of limited literature knowledge of their thermochemistry, reaction channels, and representative model N-and O-compounds [11]. Such heterogeneous composition presents a real challenge towards understanding the cross reaction processes occurring between N- and O-compounds [11, 12]. Therefore this study seeks to understand the formation of heterocyclic amines from pyrolysis of model biomass components; tyrosine and cellulose. The compounds of interest in this study include: isoindazole, 1-methylindazole, and 1-naphthyl isocyanate. Isoindazole and its derivatives are known to display a broad range of biological activities [2]. Moreover, these chemicals feature prominently in pharmaceuticals, agrochemicals, dyes, cooked food [2, 13]. 1-methylindazole is known to be an irritant and a potentially toxic organic compounds which occurs as a deep yellow viscous liquid with a very strong unpleasant odour [13, 14]. The reaction products of interest in this study were optimized using Gaussian ‘09 computational code at the density functional quantum level of theory (DFT) in conjunction with B3LYP at 3-21G basis set [11, 15] (cf. Fig. 1).

A study on the pyrolysis of military-type propellants indicated that disposal of hazardous wastes from armaments by incineration resulted in the formation of several nitrogen-containing heterocyclic aromatic compounds believed to be of serious environmental and biological health [16, 17], carcinogenic, mutagenic, and cause for possible pulmonary diseases such as cardiac arrest. Interestingly, pyrolysis of biomass has lately attracted a lot of interest from researchers because of the health, biological and environmental impacts of the intermediate radicals and molecular products of pyrolysis usually generated by such thermal degradation events including uncontrolled temperatures during cooking [18, 19]. It has been proven that N-containing heterocyclic chemicals produced during pyrolysis of proteins and amino acids are in turn metabolized most effectively to mutagenic intermediates by the form of P-450 that is induced in the form of the notorious benzo(a)pyrene [20]. Human tissues can bioactivate heterocyclic aromatic amines to produce reactive intermediates that bind to DNA to cause cellular injury and disease [20, 21].

As the concerns for energy supply and pollution problems caused by burning fossil fuels become more pronounced, mounting attention has been paid to the use of renewable and clean energy combustion of biomass materials but this shift towards cleaner fuels is not without challenges occasioned by serious environmental pollutants such as nitrogenated heterocycles [22]. Among alternatives for clean energy combustion touted to reduce environmental pollution are pyrolysis, gasification, liquefaction, and fermentation of biomass components [23]. These thermo-chemical processes convert biomass into high-value products such as bio-oil and essential oils but also release environmental pollutants to the environment [24]. With the use of computational strategies, feasible cross reactions between tyrosine and cellulose in the formation of pyrolysis products are suggested in this study.

## Materials and methods

**Fig. 2:**
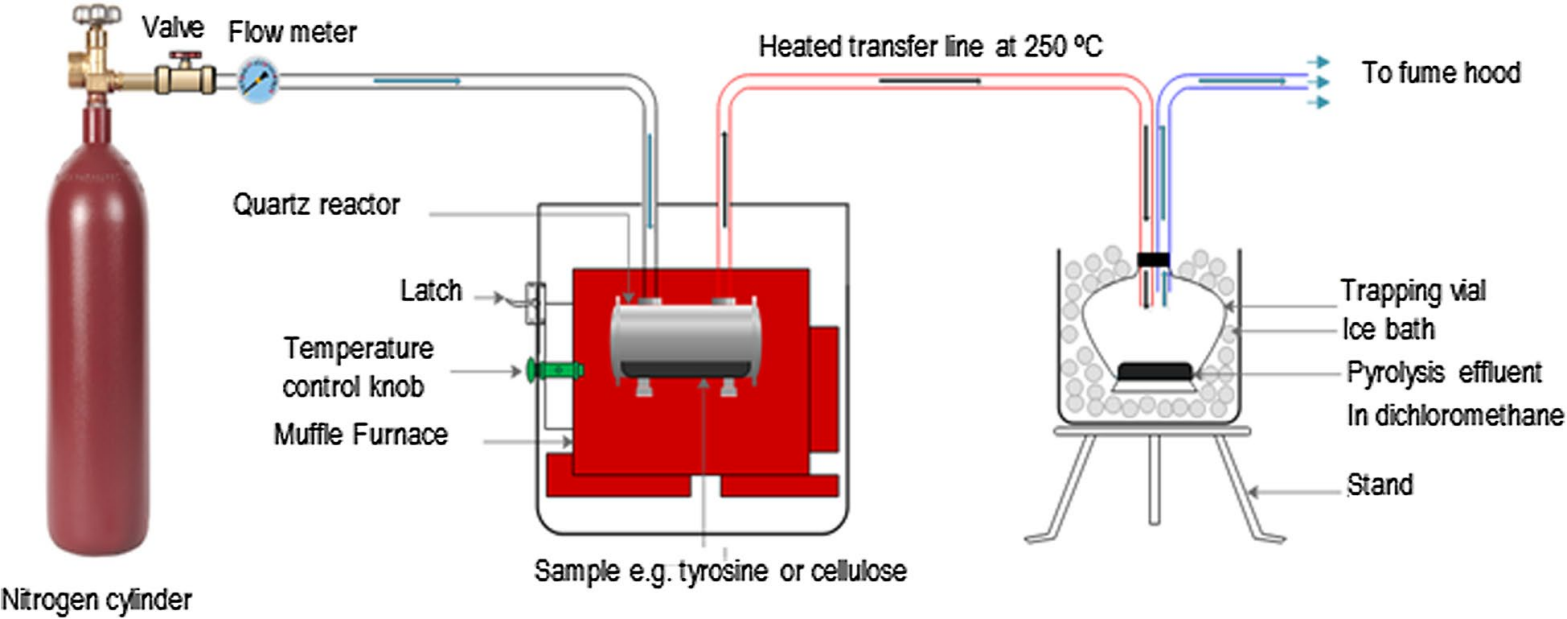
The reactor assembly and analyte collection apparatus

Tyrosine and cellulose used in this study were of analytical grade and were purchased from Sigma Aldrich Inc., (St. Louis Missouri, USA). The %purity of tyrosine was > 99 while the mesh size of cellulose was 150–200 nm. 50 ± 2 mg of equimassic mixture of cellulose by weight was packed in a tubular quartz reactor of dimensions: i.d. 0.4 cm × 17.9 cm (≈ 2.25 cm^3^ volume). The binary mixture of tyrosine and cellulose sample in the quartz reactor was placed in an electrical heater furnace whose maximum heating temperature is 1000 °C with heating rate of ~ 20 °C s^−1^ and a temperature gradient of ± 5 °C. The flow rate of the pyrolysis gas (N_2_) was set at 2.0 s. This is consistent with a gas delivery of $191 \;\text{cm}^{3} \text{min}^{ - 1}$ and $147\;\text{cm}^{3} \text{min}^{ - 1}$ for instance at 300 °C and 600 °C, respectively according to the expression reported by Kibet et al. [25]. The binary mixture was heated in flowing inert nitrogen atmosphere, and the pyrolysate was allowed to pass through a transfer column and collected in 5 mL cold dichloromethane in a conical flask (Fig. 2), and sampled into a 2 mL crimp top amber vials for GCMS analysis.

The total pyrolysis time was 3 min. This experiment was conducted under fractional pyrolysis reported elsewhere in literature [3] and the evolution of 1-methylindole, isoindazole, and 1-naphthyl isocyanate were monitored between 200 and 700 °C. A total of three replicates were conducted in this experiment. Thermal emission condensate (tar) was trapped in a 5 mL conical flask immersed in an ice bath and weighed by the method of difference. All the data reported in this study were averaged replicates of two or more data sets. The enthalpy changes reported in this study were calculated using Gaussian 09 computational platform at the density functional theory (DFT) quantum level coupled with B3LYP correlation function using the 3-21G basis set [26]. Generally, the density functional theory method combined with the relatively accurate ab initio calculations can investigate reaction mechanisms that have been postulated to be of significant importance during the thermal degradation of biomass materials [11].

### GCMS identification of pyrolysis products

GCMS analysis was carried out using an Agilent Technologies 7890A GC system coupled with a triple quadruple Agilent Technologies 5975C inert XL electron ionization/chemical ionization (EI/CI) with a triple axis mass selective detector, using a DB5-MS GC column (30 m × 0.25 mm × 0.25 µm) [27]. The temperature of the injector port was set at 250 °C to enable the conversion of organic components into the gas-phase prior to MS analysis. The carrier gas was ultra-high pure (UHP) helium (99.999%) while the flow rate of the carrier gas was set at 3.3 mL/min at 1 atmosphere pressure. Temperature programming was applied at a heating rate of 15 °C for 10 min, holding for 1 min at 200 °C, followed by a heating rate of 25 °C for 5 min, and holding for 5 min at 300 °C. The data was run through the NIST library database as an additional tool to confirm the identity of compounds [3]. The mass spectrometer was operated using Electron impact ionization energy of 70 eV in Total ion current mode (TIC) over a mass scan range of 15 amu to 600 amu. To ensure that the right compound was identified standards of purity > 99% were run through the GCMS system and the retention times compared with the compounds of interest. Calibration curves of $R^{2} > 0.98$ were constructed and the yields of evolved products with pyrolysis temperature were determined. The pyrolysis products were calibrated to obtain their yields at each pyrolysis temperature.

## Results and discussion

**Fig. 3:**
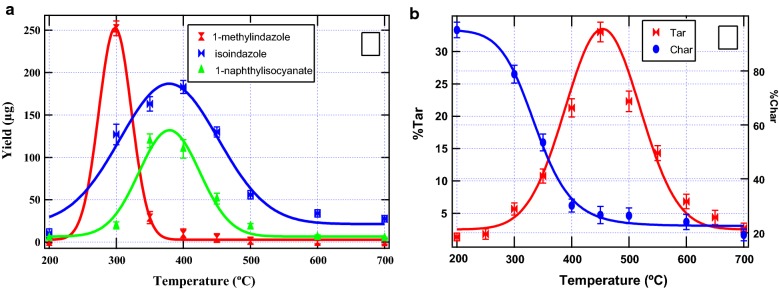
Yields of selected pyrolysis products **a** char and **b** char and tar yield as a function of pyrolysis temperature

Remarkably from this study, it was evident that cellulose based nitrogenated pyrolysis products appeared to be inhibited by tyrosine. This observation will be the focal point in the mechanistic description of the formation of nitrogenated molecular products. The quantitative release of selected evolution of nitrogenated heterocyclic molecular compounds as a function of pyrolysis temperature was explored experimentally and reported in Fig. 3a. Whereas other reaction products (isoindazole and 1-naphthyl isocyanate) reached a maximum release between 300 and 450 °C, 1-methyl indazole was evolved in high yields (250 µg) initially at 300 °C but decreased rapidly with increase in temperature. Evidently, the yield of most reaction products decreased sharply above 500 °C.

Thus at elevated temperatures, the major reaction by-product is largely carbonaceous while at lower temperatures (in this case, below 500 °C) the principle components of pyrolysis are the constituents of tar and incondensable gases such as CO_2_, CO, methane, and hydrogen [27, 28]. In the entire pyrolysis temperature, isoindazole yielded ~ 730 µg while 1-methyl isoindzaole yielded 336 µg within the same temperature range. Chemically speaking, a cooking temperature > 200 °C (Fig. 3) may yield hazardous molecular products that may be injurious to human health.

Obviously, isoindazole contributed over 50% yields of the molecular products reported in this work. It is possible to notice that 1-methylindazole may be decomposing immediately to isoindazole and probably other reaction products such as hydrogen cyanide and methane because of its apparent reactive nature (the ease to abstract a methyl radical by an H radical present in the reaction pool). This has been presented aptly in scheme 2, vide infra.

The %char yield decreased steadily between 200 and 400 °C (Fig. 3b), and approximately levelled off between 450 and 700 °C. The first initial loss from 200 to 300 °C was about 16% while the mass loss between 300 and 350 °C was ~ 25%. An approximately similar change was realized between 400 and 450 °C (24%). Clearly, 50% of the mass loss was noted in the temperature range 300 to 450 °C. Individual pyrolysis of the biomass components shows very interesting results (Table 1). For instance, the pyrolysis range 300 to 450 °C, the %char yield from the pyrolysis of individual tyrosine was 45.1 while that of pure cellulose within the same temperature range was 60.8.

**Table 1:** % char yields of individual pyrolysis of model biomass materials

Temp. (°C)	200	300	350	400	450	500	600	700
% char yield (tyrosine)	99.9	81.5	46.1	37.0	36.4	33.3	26.6	18.5
% char yield (cellulose)	92.7	91.4	90.2	32.7	30.8	27.6	21.6	21.3

Taking the average of the two biomass materials (assuming co-pyrolysis of the two components) gives ~ 50% mass loss similar to the % mass loss obtained when the two biomass materials were co-pyrolyzed. This observation is by far the most striking feature in biomass pyrolysis ever reported in literature especially because the temperature region between 300 and 450 °C is of remarkable importance in biomass pyrolysis as it coincides with the temperature range where high yields of reaction pyrolysis products are released—consistent with literature data reported elsewhere [27, 29, 30] and in this investigation (cf. Fig. 3a). To prove this hypothesis, experimental runs for the pyrolytic mixture of cellulose and tyrosine at 75:25% and 25:75% were conducted and reported in Table 2. Remarkably, the change in char yield between 300 and 450 °C for the pyrolysis of 25% tyrosine in 75% cellulose was found to be ~ 48% whereas the change in char yield for the pyrolysis of 75% tyrosine in 25% cellulose was 49%. This is consistent with the data presented in Table 1 and Fig. 3b. It was observed that the char yields across all temperatures for the two experiments did not differ significantly. The small change in mass loss between 200 and 400 °C may be attributed to observations reported elsewhere in literature [31, 32].

**Table 2:** % char yields of binary mixtures from the pyrolysis of model biomass materials

Temp. (°C)	200	300	350	400	450	500	600	700
% char yield (tyrosine: cellulose), ratio 1:3	98.3	96.4	94.2	89.2	46.6	32.8	28.5	23.2
% char yield (cellulose: tyrosine), ratio 3:1	99.1	98.1	90.0	86.4	49.3	38.1	25.3	21.7

The characteristic formation of tar in this work peaks at ≈ 450 °C (33% tar yield) as observed in Fig. 3b. At this temperature (450 °C), the % char yield was found to be 27%. From a chemical standpoint, tar is a matrix that constitutes various reaction products of pyrolysis and is a very important by-product of biomass pyrolysis since it is a major precursor in the pyrosynthesis of biofuels and essential oils [24]. Clearly, as the char yields decrease, the tar yields increase steadily from 200 to 450 °C before decreasing sharply to about 3% at 700 °C which corresponds to a char yield of ~ 19%. It can conclusively be noted that above 450 °C, both char and tar yields decrease with increasing temperature (Fig. 3b).

### Proposed mechanistic formation of reaction products

It is important to appreciate that the formation of reaction products from the co-pyrolysis of tyrosine and cellulose is an arduous task. Few studies in literature that attempt to describe the mechanistic interaction between cellulosic biomass materials and N-biomass compounds are shallow and mainly discuss probabilistic approaches rather than actual chemical interactions [33]. This study takes a critical look at previous mechanistic studies on the thermal degradation of cellulose and tyrosine and decides on possible pathways for the formation of selected molecular products. Despite years of intense research, understanding the fundamentals underlying reaction mechanisms of co-pyrolysis between cellulosic materials and N-biomass components such as tyrosine is still unclear. The suggested first step is to consider the monomeric form of cellulose from which various mechanistic processes can be intuitively derived. The thermal degradation of cellulose has been extensively studied and will not be the subject of intense discussion in this work [34, 35]. The enthalpy changes were estimated using the conventional thermodynamic Eq. 1. Accordingly, only the reaction pathways central to this study were considered.

1 $$\Delta_{r} H^{0} = \sum (\varepsilon_{0} - H_{corr} )_{products} - \sum (\varepsilon_{0} - H_{corr} )_{Reactants}$$

where, $\Delta rH^\circ$ is change in enthalpy of the reaction,$H_{corr}$ is correction to the thermal enthalpy and $\varepsilon_{0 }$ is the sum of electronic and thermal enthalpies.

**Scheme 1:**
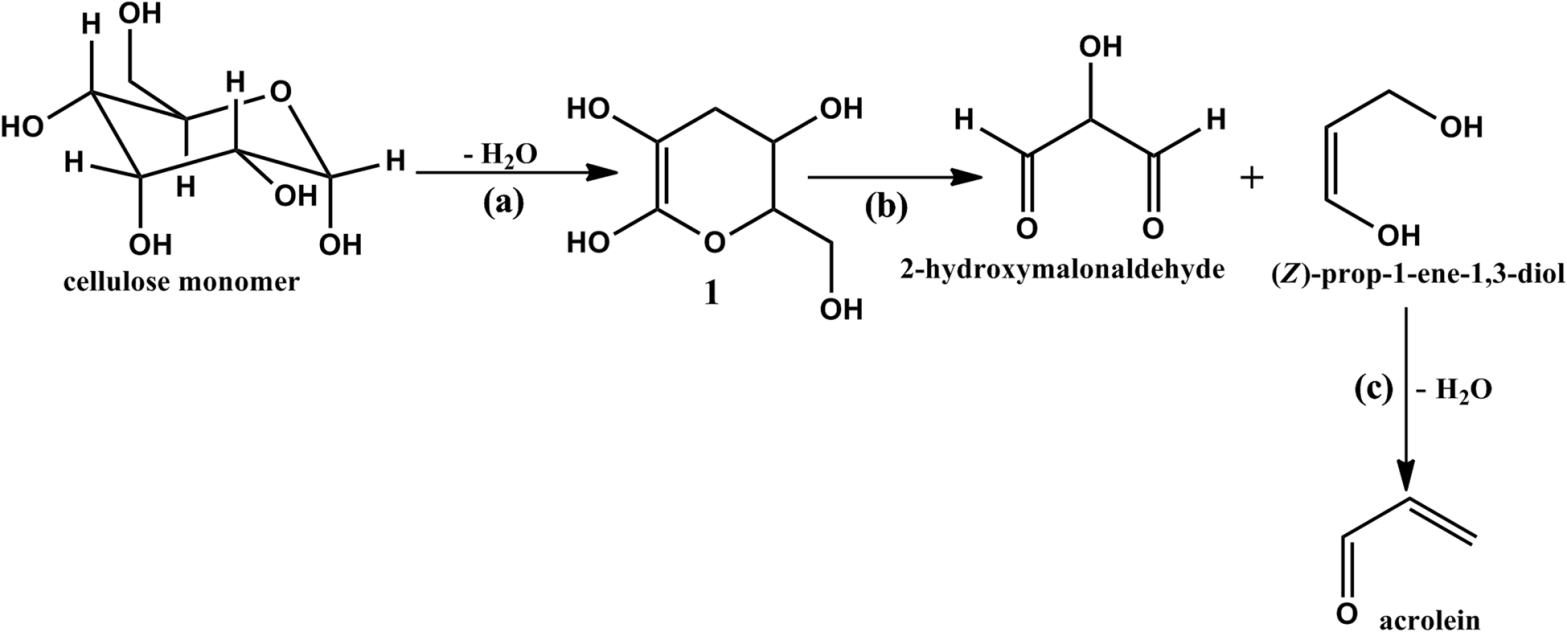
Suggested mechanism for the formation of acrolein precursor for the formation of 1-naphthyl isocyanate (Scheme 2; reaction iii, vide infra)

**Scheme 2:**
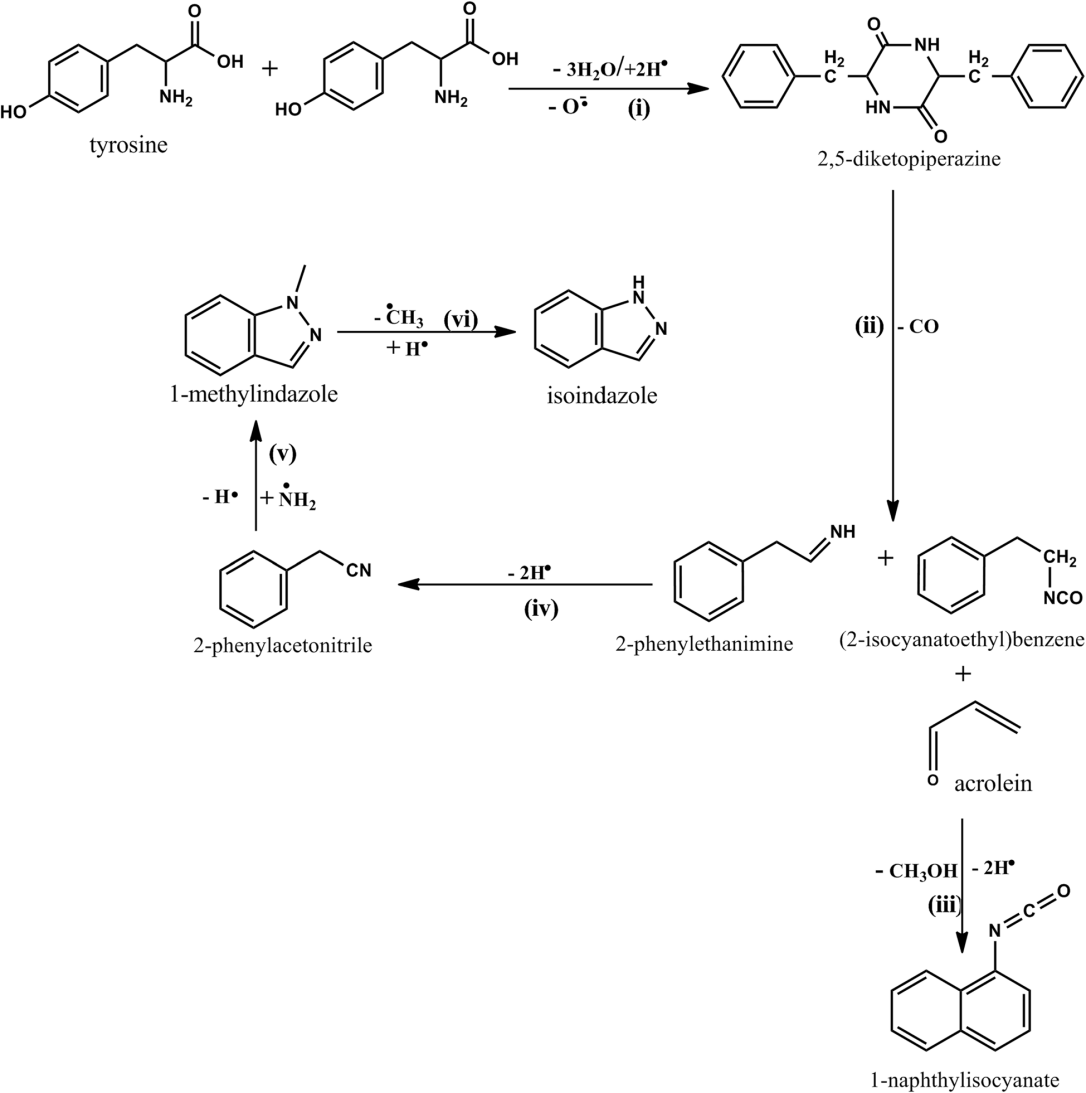
The suggested mechanistic formation of heterocyclic molecular reaction products

In this study, monomeric cellulose is proposed to undergo dehydration to form compound (**1**) [35] (Scheme 1) which may be an important precursor in the formation of acrolein considered central to this work. The reaction was found to proceed with a decent enthalpy change of $102 \;\text{kJmol}^{ - 1}$. The conversion of monomeric cellulose to compound **1** therefore takes place at fairly low energy and thus, may appear appreciably feasible in the formation of the acrolein precursor. Nevertheless, other mechanistic channels including polymerization, depolymerisation, random bond breaking and bond formation, and recombination cannot be ruled out [25, 36]. Compound 1 converts to 2-hydroxymalonaldehyde and (Z)-prop-1-ene-1,3-diol via reaction (**b**) accompanied by an enthalpy change of $167\;\text{kJmol}^{ - 1}$. On the other hand, (Z)-prop-1-ene-1,3-diol converts to acrolein via reaction (**c**) at a significantly low enthalpy change of $63\; \text{kJmol}^{ - 1}$. Although, there are many possible routes in the interaction of tyrosine and cellulose to form the observed pyrolysis products, this study explores acrolein as a major precursor in the formation of 1-naphthyl isocyanate via reaction **(iii**) in scheme 2, vide infra. Related data including structure optimization and electron density maps is presented in additional file 1.

This reaction was estimated to proceed at an enthalpy change of $426 \;\text{kJmol}^{ - 1}$. The high energy required to execute this process is consistent with the low yields of 1-naphthyl isocyanate observed in this investigation (cf. Fig. 3). Nonetheless, this study recognises that there might be other feasible mechanistic channels that result in the formation of 1-naphthyl isocyanate and therefore further studies are recommended to propose other feasible mechanistic pathways that may be added into the current body of knowledge. It is a challenging task to delve into the complicated interaction mechanism between tyrosine and cellulose without considering possible reaction pathways in the mechanistic formation of heterocyclic amines from tyrosine and taking into account that tyrosine could be inhibiting the formation of cellulose based nitrogenated heterocycles. The assumption made in this study is that cellulose which does not contain the N-reaction center may possibly be the catalyst and thus most cellulose pyrolysis products are inhibited by tyrosine. If this assumption is not made, it would be extremely complex to figure out the formation of compounds of interest reported in Fig. 3, vide supra. Accordingly, the mechanistic degradation of tyrosine is of central importance in this study following the work of Li et al. [37] which found that the major product included phenol, phenyl acetylnitrile, and 2-phenyl ethanimine. Li [37] and his co-workers postulated the decomposition pathways of tyrosine without providing the thermochemistry of its degradation channels. On the other hand, Kibet and his co-workers explored the thermal degradation of tyrosine and reported the major reaction products were phenol and it derivatives (*p*-cresol and *p*-tyramine)—no thermochemistry were also provided in this study [3]. Consequently, this study has identified these gaps and estimated the relevant thermochemical values corresponding to the formation of the thermal by-products of interest as suggested in Scheme 2.

The first step in the degradation of tyrosine is the formation of the intermediate 2,5-diketopiperazine. This step involves the combination of two tyrosine molecules via reaction (**i**) accompanied by an enthalpy change of $248 \;\text{kJmol}^{ - 1}$. The short lived intermediate [33] is suggested to degrade thermally accompanied by the loss of a $\text{CO}$ via reaction (**ii**) to form two major precursor species; 2-phenylethanimine and (2-isocyanoethyl)benzene. This reaction proceeds with an enthalpy of $256 \;\text{kJmol}^{ - 1}$. This work proposes that (2-isocyanoethyl)benzene reacts with acrolein possibly from cellulose via reaction (**iii**) to form one of the most hazardous compounds (1-naphthyl isocyanate as reported in scheme 2, vide supra) believed to be a major cause of respiratory disorders [4, 5].

Besides, 2-phenylethanimine degrades via reaction (**iv**) to form phenylacetonitrile which is considered a major progenitor for the formation of 1-methylindazole. Reaction (**iv**) takes place with a modest enthalpy change of $- \;55 \;\text{kJmol}^{ - 1}$. The formation of 1-methylindazole via reaction (**v**) occurs with an enthalpy change of $- \;59\, \text{kJmol}^{ - 1}$. The conversion of 1-methylindazole to isoindazole according to reaction (**vi**) occurs via the scission of a methyl $\left( {\text{CH}_{3} } \right)$ accompanied by an enthalpy change of $- \;178 \;\text{kJmol}^{ - 1}$. This energy is significantly high suggesting that there might be other routes that may lead to the formation of isoindazole.

Alternative mechanistic pathways that can results in the formation of isoindazole, 1-methyl indazole, and 1-naphthyl isocyanate may include dehydration, decarboxylation, bond rapture, concerted reactions, random bond breaking and rearrangement. The proposed reaction mechanism underlines a plausible basis for further mechanistic studies involving the interaction of N-biomass components with cellulosic materials in striking a balance between environmental safety and clean energy combustion. This work therefore while concerned about the interaction of tyrosine with cellulose during high temperature cooking provides estimated thermochemical energies involved in the formation of nitrogenated compounds considered detrimental to both environmental and public health.

## Conclusion

This work has explored extensively the formation of selected hazardous heterocyclic amines from the co-pyrolysis of model biomass materials; tyrosine and cellulose. Possible mechanistic pathways during the pyrolytic interaction of tyrosine and cellulose to form nitrogenated reaction products believed to be of grave concern to human health during high temperature pyrolysis of binary biomass components are suggested. The proposed mechanistic channel was biased towards selective formation of molecular products from the interaction of amino acid components and cellulose during thermolysis processes in which tyrosine inhibited cellulose based reaction products. Isoindazole was noted as the major pyrolysis product accounting for over 50% of the heterocyclic products reported in this work. Notably, 1-naphthyl isocyanate established as a serious health toxicant was also formed in significant amounts (~ 25% of the total products reported). Thus, the evolution of heterocyclic amines from the co-pyrolysis of tyrosine and cellulose provides an insight into the combustion events of numerous origins (municipal incineration, biofuel production, fireworks, and explosives) considered detrimental to human and environmental health. Since most explosives used in war release hazardous heterocyclic amines, it has become inevitably necessary to champion peace and avoid war as much as possible in the interest of public health and loss of life. The greatest mass loss from the co-pyrolysis of the model biomass components investigated was observed between 300 and 450 °C. This is in line with other studies reported in literature. More importantly, proper combustion practices may reduce the medical budget in line with millennium development goals.

## Supplementary information

**Additional file 1.** Raw data for char yields, tar yields, concentrations of compounds of interest, and optimized structures of the heterocyclic structures central to enthalpy changes. Raw thermochemistry data and stationary points for isoindazole and 1-methyl indazole.

## Abbreviations

$\Delta rH^\circ$: is change in enthalpy of the reaction
$H_{corr}$: is correction to the thermal enthalpy
$\varepsilon_{0 }$: is the sum of electronic and thermal enthalpies
EI/CI: electron ionization/chemical ionization
DFT: density functional theory
B3LYP: Becke, 3-parameter, Lee–Yang–Parr
