# Supplementary material for: Mechanistic formation of hazardous molecular heterocyclic amines from high temperature pyrolysis of model biomass materials: cellulose and tyrosine
Source: BMC Chem. 2019 Nov 8;13(1):126. doi: 10.1186/s13065-019-0644-1 (PMC6842190; doi:10.1186/s13065-019-0644-1)
Supplement: Supplementary file 1 — Additional file 1. Raw data for char yields, tar yields, concentrations of compounds of interest, and optimized structures of the heterocyclic structures central to enthalpy changes. Raw thermochemistry data and stationary points for isoindazole and 1-methyl indazole. [file 13065_2019_644_MOESM1_ESM.docx]

**Mechanistic formation of hazardous molecular heterocyclic amines from high temperature pyrolysis of model biomass materials: cellulose and tyrosine**

Samuel K. Kirkok^1^, Joshua K. Kibet^1^*, Francis Okanga^1^, Thomas Kinyanjui^1^, Vincent Nyamori^2^

^1^Department of Chemistry, Egerton University, P.O Box 536 -20115, Egerton

^2^School of Chemistry and Physics, University of KwaZulu-Natal, Westville Campus, Private Bag X54001, Durban 4000, South Africa

Author for correspondence: [jkibet@egerton.ac.ke](mailto:jkibet@egerton.ac.ke)

**Additional Supporting Information**

**S1. Tar and Char Data**

| Temp.  ($^{\circ}C)$ | % Yield | | | | |
| --- | --- | --- | --- | --- | --- |
|  | Tar | Error Tar | Temp. 2 | Char | Error Char |
| 200 | 1.34 | 0.6 | 200 | 95.2 | 2.9 |
| 250 | 1.79 | 0.8 | 300 | 78.8 | 3.3 |
| 300 | 5.68 | 0.95 | 350 | 53.5 | 3.1 |
| 350 | 10.75 | 1.1 | 400 | 30.1 | 2.4 |
| 400 | 21.29 | 1.4 | 450 | 26.6 | 3.2 |
| 450 | 33.04 | 1.5 | 500 | 26.3 | 2.9 |
| 500 | 22.32 | 1.6 | 600 | 24 | 2.8 |
| 550 | 14.29 | 1.15 | 700 | 19.2 | 2.2 |
| 600 | 6.82 | 1.12 |  |  |  |
| 650 | 4.36 | 1.1 |  |  |  |
| 700 | 2.68 | 0.8 |  |  |  |
| 750 | 1.79 | 0.2 |  |  |  |

**S2: % Yields of Tar and Char**

| Temp.  ($^{\circ}C)$ | Conc. (ug) | | | | | | % Yield | |
| --- | --- | --- | --- | --- | --- | --- | --- | --- |
|  | isoindazole | Error | 1-methyl indazole | Error | 1-naphthyl isocyanate | Error | Char Yield | Error |
| 200 | 10.55 | 1.5 | 0.64 | 0.2 | 5.05 | 1.3 | 95.2 | 2.9 |
| 300 | 127 | 4.1 | 252 | 8.8 | 19.7 | 3.2 | 78.8 | 3.3 |
| 350 | 163 | 8.2 | 29.15 | 3.1 | 119.5 | 8.5 | 53.5 | 3.1 |
| 400 | 183 | 10.8 | 9.8 | 1.8 | 110 | 7.8 | 30.1 | 2.4 |
| 450 | 130 | 6.3 | 4.87 | 1.2 | 51.5 | 5.92 | 26.6 | 3.2 |
| 500 | 56 | 3.1 | 1.74 | 0.92 | 18.8 | 2.91 | 26.3 | 2.9 |
| 600 | 34 | 2.6 | 0.63 | 0.15 | 6.1 | 1.45 | 24 | 2.8 |
| 700 | 27.65 | 1.25 | 0.161 | 0.4 | 4.885 | 0.8 | 19.2 | 2.2 |

**S3: Input and Optimized molecular structures of compounds of interest**


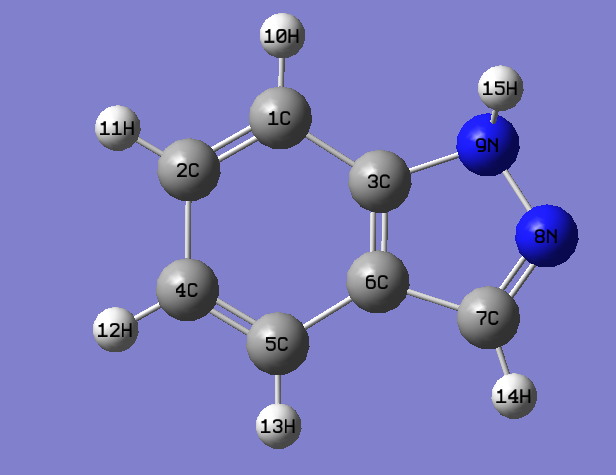


Input structure for Isoindazole


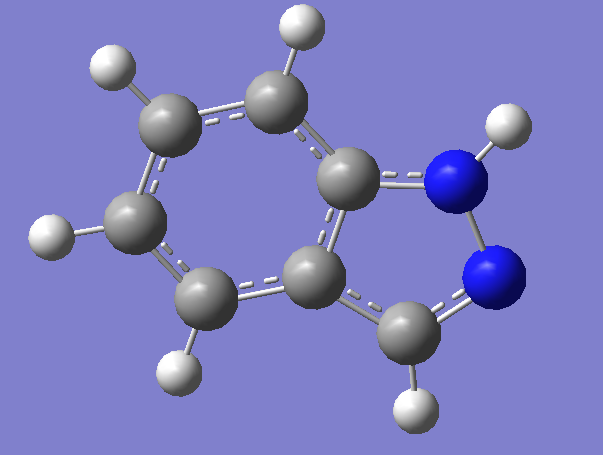


Optimized structure for isoindazole


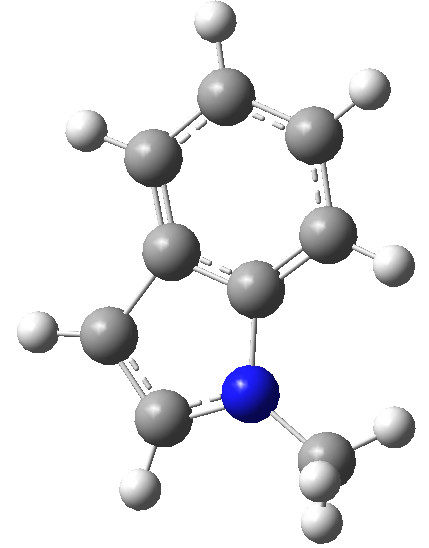


Input structure for 1-methyl indole


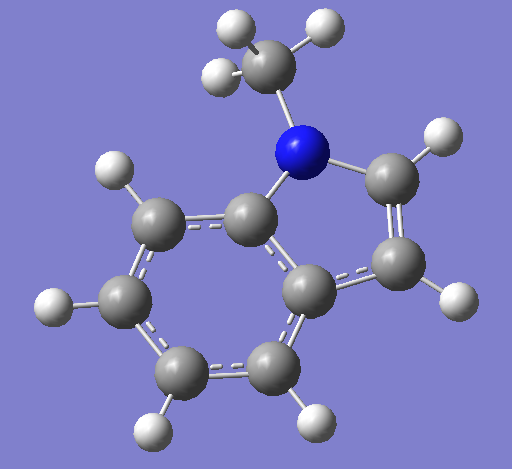


Optimized structure for 1-methyl indole


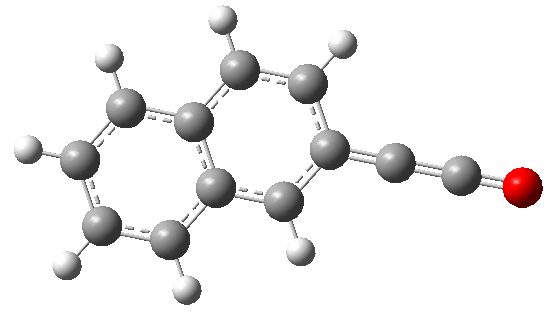


Input structure of 1-naphthylisocyanate


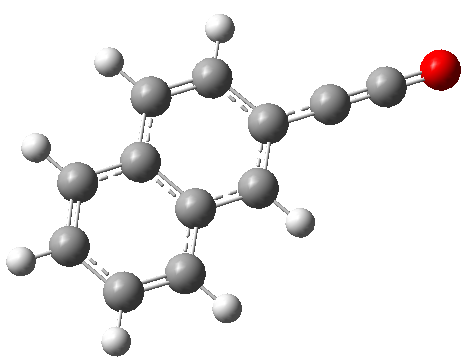


Optimized structure of 1-naphthylisocyanate

**S4: Molecular orbital diagrams for compounds of interest**


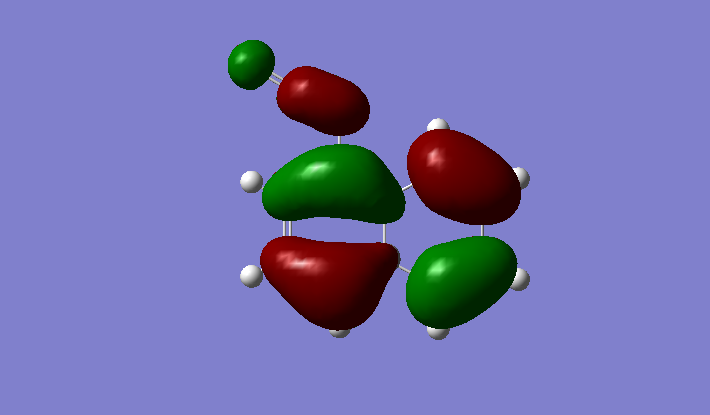


1-naphthylisocyanate


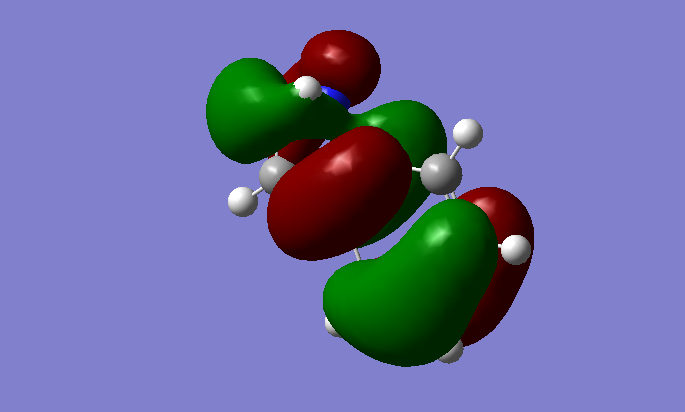


Isoindazole


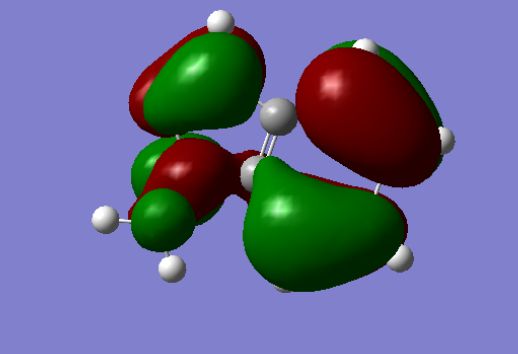


1-methylindazole

**S5: Example of optimization Energies**

1. **Isoindole**

-------------------

- Thermochemistry -

-------------------

Temperature 298.150 Kelvin. Pressure 1.00000 Atm.

Atom 1 has atomic number 6 and mass 12.00000

Atom 2 has atomic number 6 and mass 12.00000

Atom 3 has atomic number 6 and mass 12.00000

Atom 4 has atomic number 6 and mass 12.00000

Atom 5 has atomic number 6 and mass 12.00000

Atom 6 has atomic number 6 and mass 12.00000

Atom 7 has atomic number 6 and mass 12.00000

Atom 8 has atomic number 1 and mass 1.00783

Atom 9 has atomic number 1 and mass 1.00783

Atom 10 has atomic number 1 and mass 1.00783

Atom 11 has atomic number 1 and mass 1.00783

Atom 12 has atomic number 1 and mass 1.00783

Atom 13 has atomic number 1 and mass 1.00783

Atom 14 has atomic number 7 and mass 14.00307

Atom 15 has atomic number 7 and mass 14.00307

Molecular mass: 118.05310 amu.

Principal axes and moments of inertia in atomic units:

1 2 3

EIGENVALUES -- 457.164021116.971451574.13544

X 1.00000 0.00161 0.00000

Y -0.00161 1.00000 -0.00001

Z 0.00000 0.00001 1.00000

This molecule is an asymmetric top.

Rotational symmetry number 1.

Warning -- assumption of classical behavior for rotation

may cause significant error

Rotational temperatures (Kelvin) 0.18946 0.07754 0.05502

Rotational constants (GHZ): 3.94769 1.61575 1.14650

Zero-point vibrational energy 312160.9 (Joules/Mol)

74.60825 (Kcal/Mol)

Warning -- explicit consideration of 7 degrees of freedom as

vibrations may cause significant error

Vibrational temperatures: 319.19 377.35 584.79 649.03 771.32

(Kelvin) 803.65 865.32 929.17 951.91 1115.15

1120.61 1173.73 1263.03 1321.61 1331.39

1343.84 1415.24 1452.64 1476.61 1498.61

1679.11 1740.06 1782.01 1840.13 1857.39

1885.13 1981.70 2034.73 2048.67 2183.23

2215.54 2305.24 2373.00 4596.21 4607.50

4623.56 4637.89 4718.16 5215.13

Zero-point correction= 0.118896 (Hartree/Particle)

Thermal correction to Energy= 0.124809

Thermal correction to Enthalpy= 0.125753

Thermal correction to Gibbs Free Energy= 0.088779

Sum of electronic and zero-point Energies= -377.623299

Sum of electronic and thermal Energies= -377.617386

Sum of electronic and thermal Enthalpies= -377.616441

Sum of electronic and thermal Free Energies= -377.653415

E (Thermal) CV S

KCal/Mol Cal/Mol-Kelvin Cal/Mol-Kelvin

Total 78.319 24.470 77.818

Electronic 0.000 0.000 0.000

Translational 0.889 2.981 40.213

Rotational 0.889 2.981 28.177

Vibrational 76.541 18.509 9.429

Vibration 1 0.648 1.808 1.944

Vibration 2 0.670 1.742 1.647

Vibration 3 0.771 1.456 0.939

Vibration 4 0.810 1.358 0.792

Vibration 5 0.891 1.170 0.574

Vibration 6 0.914 1.121 0.527

Vibration 7 0.960 1.029 0.447

Q Log10(Q) Ln(Q)

Total Bot 0.146023D-40 -40.835579 -94.027396

Total V=0 0.712173D+14 13.852585 31.896757

Vib (Bot) 0.902454D-54 -54.044575 -124.442233

Vib (Bot) 1 0.890927D+00 -0.050158 -0.115492

Vib (Bot) 2 0.739750D+00 -0.130915 -0.301443

Vib (Bot) 3 0.436438D+00 -0.360078 -0.829110

Vib (Bot) 4 0.379817D+00 -0.420425 -0.968065

Vib (Bot) 5 0.296628D+00 -0.527788 -1.215276

Vib (Bot) 6 0.278644D+00 -0.554951 -1.277822

Vib (Bot) 7 0.247910D+00 -0.605706 -1.394690

Vib (V=0) 0.440139D+01 0.643590 1.481920

Vib (V=0) 1 0.152164D+01 0.182312 0.419790

Vib (V=0) 2 0.139288D+01 0.143913 0.331372

Vib (V=0) 3 0.116368D+01 0.065835 0.151592

Vib (V=0) 4 0.112790D+01 0.052271 0.120359

Vib (V=0) 5 0.108137D+01 0.033973 0.078227

Vib (V=0) 6 0.107240D+01 0.030357 0.069900

Vib (V=0) 7 0.105809D+01 0.024521 0.056461

Electronic 0.100000D+01 0.000000 0.000000

Translational 0.504163D+08 7.702571 17.735826

Rotational 0.320940D+06 5.506424 12.679011

***** Axes restored to original set *****

-------------------------------------------------------------------

Center Atomic Forces (Hartrees/Bohr)

Number Number X Y Z

-------------------------------------------------------------------

1 6 -0.000092853 -0.000025933 0.000085968

2 6 -0.000037442 -0.000147501 -0.000086134

3 6 -0.000000889 0.000027959 -0.000045994

4 6 -0.000030606 -0.000006079 0.000016402

5 6 0.000022403 0.000002201 0.000014662

6 6 -0.000005426 -0.000006847 -0.000013920

7 6 -0.000038162 -0.000054237 0.000140762

8 1 0.000010668 0.000000195 0.000010953

9 1 0.000004940 0.000009089 0.000000525

10 1 -0.000005361 0.000007416 -0.000005159

11 1 -0.000010566 0.000009105 -0.000000866

12 1 0.000044378 0.000047200 -0.000010898

13 1 0.000000693 -0.000011559 -0.000019510

14 7 0.000102761 0.000120959 -0.000078188

15 7 0.000035463 0.000028034 -0.000008603

-------------------------------------------------------------------

Cartesian Forces: Max 0.000147501 RMS 0.000050664

GradGradGradGradGradGradGradGradGradGradGradGradGradGradGradGradGradGrad

Berny optimization.

Internal Forces: Max 0.000137168 RMS 0.000029317

Search for a local minimum.

Step number 1 out of a maximum of 2

All quantities printed in internal units (Hartrees-Bohrs-Radians)

Second derivative matrix not updated -- analytic derivatives used.

Eigenvalues --- 0.00707 0.01188 0.01734 0.01759 0.01985

Eigenvalues --- 0.02178 0.02410 0.02567 0.03163 0.03234

Eigenvalues --- 0.04132 0.05556 0.10799 0.11286 0.11577

Eigenvalues --- 0.11868 0.12991 0.13234 0.16730 0.19700

Eigenvalues --- 0.20292 0.20814 0.23710 0.25790 0.30039

Eigenvalues --- 0.34632 0.35931 0.36266 0.36502 0.36624

Eigenvalues --- 0.37781 0.38677 0.39997 0.40238 0.45619

Eigenvalues --- 0.46906 0.47430 0.50225 0.512801000.00000

Eigenvalues --- 1000.000001000.000001000.000001000.000001000.00000

Eigenvalues --- 1000.000001000.000001000.000001000.000001000.00000

Eigenvalues --- 1000.000001000.000001000.000001000.000001000.00000

Eigenvalues --- 1000.000001000.000001000.000001000.000001000.00000

Eigenvalues --- 1000.000001000.000001000.000001000.000001000.00000

Eigenvalues --- 1000.000001000.000001000.000001000.000001000.00000

Eigenvalues --- 1000.000001000.000001000.000001000.000001000.00000

Eigenvalues --- 1000.000001000.00000

Angle between quadratic step and forces= 56.07 degrees.

Linear search not attempted -- first point.

Iteration 1 RMS(Cart)= 0.00018610 RMS(Int)= 0.00000006

Iteration 2 RMS(Cart)= 0.00000005 RMS(Int)= 0.00000005

Variable Old X -DE/DX Delta X Delta X Delta X New X

(Linear) (Quad) (Total)

R1 2.69437 0.00003 0.00000 0.00013 0.00013 2.69450

R2 2.65071 0.00001 0.00000 0.00000 0.00000 2.65071

R3 2.59237 -0.00012 0.00000 -0.00023 -0.00023 2.59214

R4 2.65234 0.00003 0.00000 0.00008 0.00008 2.65242

R5 2.70823 -0.00003 0.00000 -0.00009 -0.00009 2.70814

R6 2.62056 0.00001 0.00000 0.00002 0.00002 2.62058

R7 2.04801 0.00001 0.00000 0.00003 0.00003 2.04804

R8 2.67335 -0.00002 0.00000 -0.00006 -0.00006 2.67329

R9 2.04717 0.00001 0.00000 0.00003 0.00003 2.04721

R10 2.62177 0.00000 0.00000 0.00001 0.00001 2.62178

R11 2.04819 0.00001 0.00000 0.00002 0.00002 2.04821

R12 2.04654 0.00001 0.00000 0.00003 0.00003 2.04657

R13 2.03578 0.00001 0.00000 0.00002 0.00002 2.03579

R14 2.52124 -0.00014 0.00000 -0.00025 -0.00025 2.52098

R15 1.90755 -0.00006 0.00000 -0.00012 -0.00012 1.90743

R16 2.66669 0.00000 0.00000 -0.00009 -0.00009 2.66660

A1 2.11548 0.00003 0.00000 0.00009 0.00009 2.11557

A2 1.85204 -0.00006 0.00000 -0.00016 -0.00016 1.85188

A3 2.31566 0.00002 0.00000 0.00008 0.00008 2.31574

A4 2.09462 -0.00004 0.00000 -0.00015 -0.00015 2.09448

A5 1.83019 0.00004 0.00000 0.00011 0.00011 1.83030

A6 2.35837 0.00000 0.00000 0.00004 0.00004 2.35841

A7 2.06974 0.00001 0.00000 0.00004 0.00004 2.06978

A8 2.10369 0.00000 0.00000 0.00000 0.00000 2.10369

A9 2.10975 0.00000 0.00000 -0.00003 -0.00003 2.10972

A10 2.11043 0.00002 0.00000 0.00005 0.00005 2.11048

A11 2.09366 -0.00001 0.00000 -0.00004 -0.00004 2.09362

A12 2.07909 -0.00001 0.00000 -0.00002 -0.00002 2.07908

A13 2.12275 0.00000 0.00000 -0.00002 -0.00002 2.12273

A14 2.07882 0.00000 0.00000 0.00002 0.00002 2.07884

A15 2.08161 0.00000 0.00000 0.00000 0.00000 2.08161

A16 2.05335 -0.00001 0.00000 -0.00002 -0.00002 2.05334

A17 2.11952 0.00001 0.00000 0.00006 0.00006 2.11958

A18 2.11031 0.00000 0.00000 -0.00005 -0.00005 2.11026

A19 2.23012 0.00003 0.00000 0.00012 0.00012 2.23024

A20 1.95857 -0.00005 0.00000 -0.00013 -0.00013 1.95844

A21 2.09450 0.00002 0.00000 0.00001 0.00001 2.09451

A22 1.82861 0.00005 0.00000 0.00011 0.00011 1.82872

A23 2.26004 -0.00004 0.00000 -0.00027 -0.00027 2.25976

A24 1.95537 0.00002 0.00000 0.00007 0.00007 1.95544

A25 2.06778 0.00002 0.00000 0.00020 0.00020 2.06798

D1 -0.00010 0.00000 0.00000 0.00010 0.00010 0.00000

D2 -3.14134 -0.00001 0.00000 -0.00025 -0.00025 3.14159

D3 -3.14132 -0.00002 0.00000 -0.00027 -0.00027 -3.14159

D4 0.00062 -0.00003 0.00000 -0.00062 -0.00062 0.00000

D5 0.00026 -0.00001 0.00000 -0.00026 -0.00026 0.00000

D6 -3.14137 -0.00001 0.00000 -0.00022 -0.00022 3.14159

D7 3.14137 0.00001 0.00000 0.00022 0.00022 3.14159

D8 -0.00026 0.00001 0.00000 0.00026 0.00026 0.00000

D9 3.14104 0.00002 0.00000 0.00056 0.00056 -3.14159

D10 -0.00024 0.00001 0.00000 0.00024 0.00024 0.00000

D11 -0.00012 0.00000 0.00000 0.00012 0.00012 0.00000

D12 -3.14140 -0.00001 0.00000 -0.00020 -0.00020 3.14159

D13 -0.00013 0.00001 0.00000 0.00013 0.00013 0.00000

D14 -3.14126 -0.00001 0.00000 -0.00033 -0.00033 3.14159

D15 3.14098 0.00002 0.00000 0.00061 0.00061 -3.14159

D16 -0.00015 0.00001 0.00000 0.00015 0.00015 0.00000

D17 -3.14122 0.00000 0.00000 -0.00037 -0.00037 3.14159

D18 -0.00085 0.00004 0.00000 0.00085 0.00085 0.00000

D19 0.00080 -0.00002 0.00000 -0.00080 -0.00080 0.00000

D20 3.14117 0.00003 0.00000 0.00042 0.00042 3.14159

D21 0.00019 -0.00001 0.00000 -0.00019 -0.00019 0.00000

D22 -3.14146 -0.00001 0.00000 -0.00013 -0.00013 -3.14159

D23 3.14132 0.00001 0.00000 0.00027 0.00027 -3.14159

D24 -0.00033 0.00001 0.00000 0.00033 0.00033 0.00000

D25 -0.00002 0.00000 0.00000 0.00002 0.00002 0.00000

D26 3.14136 0.00001 0.00000 0.00023 0.00023 -3.14159

D27 -3.14156 0.00000 0.00000 -0.00003 -0.00003 3.14159

D28 -0.00018 0.00001 0.00000 0.00018 0.00018 0.00000

D29 -0.00020 0.00001 0.00000 0.00020 0.00020 0.00000

D30 3.14143 0.00001 0.00000 0.00016 0.00016 -3.14159

D31 -3.14158 0.00000 0.00000 -0.00001 -0.00001 3.14159

D32 0.00005 0.00000 0.00000 -0.00005 -0.00005 0.00000

D33 0.00070 -0.00004 0.00000 -0.00070 -0.00070 0.00000

D34 3.14118 0.00001 0.00000 0.00042 0.00042 -3.14159

D35 -0.00028 0.00002 0.00000 0.00028 0.00028 0.00000

D36 3.14159 0.00001 0.00000 0.00000 0.00000 3.14159

Item Value Threshold Converged?

Maximum Force 0.000137 0.000450 YES

RMS Force 0.000029 0.000300 YES

Maximum Displacement 0.000813 0.001800 YES

RMS Displacement 0.000186 0.001200 YES

Predicted change in Energy=-1.602428D-07

Optimization completed.

-- Stationary point found.

**b) 1-methyl indazole**

-------------------

- Thermochemistry -

-------------------

Temperature 298.150 Kelvin. Pressure 1.00000 Atm.

Atom 1 has atomic number 6 and mass 12.00000

Atom 2 has atomic number 6 and mass 12.00000

Atom 3 has atomic number 6 and mass 12.00000

Atom 4 has atomic number 6 and mass 12.00000

Atom 5 has atomic number 6 and mass 12.00000

Atom 6 has atomic number 6 and mass 12.00000

Atom 7 has atomic number 6 and mass 12.00000

Atom 8 has atomic number 1 and mass 1.00783

Atom 9 has atomic number 1 and mass 1.00783

Atom 10 has atomic number 1 and mass 1.00783

Atom 11 has atomic number 1 and mass 1.00783

Atom 12 has atomic number 1 and mass 1.00783

Atom 13 has atomic number 7 and mass 14.00307

Atom 14 has atomic number 7 and mass 14.00307

Atom 15 has atomic number 6 and mass 12.00000

Atom 16 has atomic number 1 and mass 1.00783

Atom 17 has atomic number 1 and mass 1.00783

Atom 18 has atomic number 1 and mass 1.00783

Molecular mass: 132.06875 amu.

Principal axes and moments of inertia in atomic units:

1 2 3

EIGENVALUES -- 647.583651394.177462030.52179

X 0.99922 -0.03950 0.00000

Y 0.03950 0.99922 0.00000

Z 0.00000 0.00000 1.00000

This molecule is an asymmetric top.

Rotational symmetry number 1.

Warning -- assumption of classical behavior for rotation

may cause significant error

Rotational temperatures (Kelvin) 0.13375 0.06213 0.04266

Rotational constants (GHZ): 2.78689 1.29448 0.88881

Zero-point vibrational energy 414007.9 (Joules/Mol)

98.95026 (Kcal/Mol)

Warning -- explicit consideration of 8 degrees of freedom as

vibrations may cause significant error

Vibrational temperatures: 120.02 218.15 365.83 376.84 458.09

(Kelvin) 728.70 739.70 840.18 928.81 947.40

1027.71 1140.63 1197.23 1261.05 1323.56

1449.05 1457.09 1483.58 1524.72 1580.26

1635.76 1700.41 1757.27 1796.95 1823.23

1841.57 1918.54 1941.76 1989.86 2063.17

2114.65 2173.82 2263.32 2325.28 2357.98

2396.10 2400.27 2424.84 2495.38 2571.87

4605.98 4677.49 4783.38 4826.20 4838.57

4861.87 4875.42 4957.77

Zero-point correction= 0.157687 (Hartree/Particle)

Thermal correction to Energy= 0.164826

Thermal correction to Enthalpy= 0.165770

Thermal correction to Gibbs Free Energy= 0.125430

Sum of electronic and zero-point Energies= -413.988195

Sum of electronic and thermal Energies= -413.981056

Sum of electronic and thermal Enthalpies= -413.980112

Sum of electronic and thermal Free Energies= -414.020452

E (Thermal) CV S

KCal/Mol Cal/Mol-Kelvin Cal/Mol-Kelvin

Total 103.430 27.183 84.902

Electronic 0.000 0.000 0.000

Translational 0.889 2.981 40.547

Rotational 0.889 2.981 28.996

Vibrational 101.652 21.221 15.359

Vibration 1 0.600 1.961 3.809

Vibration 2 0.619 1.901 2.652

Vibration 3 0.665 1.756 1.701

Vibration 4 0.669 1.743 1.649

Vibration 5 0.705 1.638 1.318

Vibration 6 0.862 1.236 0.642

Vibration 7 0.869 1.219 0.624

Vibration 8 0.941 1.066 0.478

Q Log10(Q) Ln(Q)

Total Bot 0.202388D-57 -57.693816 -132.844920

Total V=0 0.687298D+15 14.837145 34.163789

Vib (Bot) 0.699958D-71 -71.154928 -163.840277

Vib (Bot) 1 0.246752D+01 0.392261 0.903215

Vib (Bot) 2 0.133673D+01 0.126044 0.290227

Vib (Bot) 3 0.766034D+00 -0.115752 -0.266529

Vib (Bot) 4 0.740867D+00 -0.130260 -0.299935

Vib (Bot) 5 0.590982D+00 -0.228426 -0.525970

Vib (Bot) 6 0.322638D+00 -0.491285 -1.131225

Vib (Bot) 7 0.315654D+00 -0.500788 -1.153107

Vib (Bot) 8 0.259912D+00 -0.585174 -1.347414

Vib (V=0) 0.237702D+02 1.376033 3.168432

Vib (V=0) 1 0.301767D+01 0.479672 1.104485

Vib (V=0) 2 0.192718D+01 0.284923 0.656059

Vib (V=0) 3 0.141477D+01 0.150686 0.346968

Vib (V=0) 4 0.139380D+01 0.144201 0.332036

Vib (V=0) 5 0.127412D+01 0.105210 0.242255

Vib (V=0) 6 0.109506D+01 0.039438 0.090808

Vib (V=0) 7 0.109130D+01 0.037945 0.087371

Vib (V=0) 8 0.106352D+01 0.026745 0.061584

Electronic 0.100000D+01 0.000000 0.000000

Translational 0.596562D+08 7.775655 17.904108

Rotational 0.484682D+06 5.685457 13.091249

***** Axes restored to original set *****

-------------------------------------------------------------------

Center Atomic Forces (Hartrees/Bohr)

Number Number X Y Z

-------------------------------------------------------------------

1 6 -0.000038148 -0.000062210 -0.000006627

2 6 0.000017516 -0.000034242 -0.000010342

3 6 0.000022241 0.000009021 0.000026979

4 6 -0.000006401 0.000003300 0.000002165

5 6 0.000015731 -0.000018517 -0.000031063

6 6 0.000005627 0.000037546 0.000046580

7 6 -0.000000789 -0.000001688 -0.000022978

8 1 -0.000002441 0.000000966 -0.000006960

9 1 -0.000000048 0.000002668 -0.000002833

10 1 -0.000000841 0.000002337 0.000001724

11 1 0.000003355 0.000000631 -0.000005712

12 1 -0.000004251 -0.000002813 0.000002820

13 7 -0.000031722 0.000005818 -0.000019778

14 7 0.000009870 0.000027224 0.000013123

15 6 0.000012204 0.000030798 0.000013081

16 1 -0.000006362 0.000004252 -0.000004669

17 1 -0.000003595 -0.000000685 -0.000001412

18 1 0.000008055 -0.000004404 0.000005903

-------------------------------------------------------------------

Cartesian Forces: Max 0.000062210 RMS 0.000018307

GradGradGradGradGradGradGradGradGradGradGradGradGradGradGradGradGradGrad

Berny optimization.

Internal Forces: Max 0.000047495 RMS 0.000011287

Search for a local minimum.

Step number 1 out of a maximum of 2

All quantities printed in internal units (Hartrees-Bohrs-Radians)

Second derivative matrix not updated -- analytic derivatives used.

Eigenvalues --- 0.00052 0.00720 0.01452 0.02120 0.02193

Eigenvalues --- 0.02442 0.02785 0.03051 0.03244 0.03912

Eigenvalues --- 0.04051 0.04768 0.06250 0.07479 0.07573

Eigenvalues --- 0.12602 0.13093 0.13572 0.14714 0.14988

Eigenvalues --- 0.15302 0.16489 0.19651 0.21445 0.21888

Eigenvalues --- 0.23165 0.23648 0.24943 0.26690 0.27492

Eigenvalues --- 0.32274 0.36661 0.37271 0.39010 0.39424

Eigenvalues --- 0.39866 0.40102 0.40278 0.40461 0.41288

Eigenvalues --- 0.42427 0.43443 0.44857 0.45376 0.53392

Eigenvalues --- 0.54742 0.57564 0.630851000.000001000.00000

Eigenvalues --- 1000.000001000.000001000.000001000.000001000.00000

Eigenvalues --- 1000.000001000.000001000.000001000.000001000.00000

Eigenvalues --- 1000.000001000.000001000.000001000.000001000.00000

Eigenvalues --- 1000.000001000.000001000.000001000.000001000.00000

Eigenvalues --- 1000.000001000.000001000.000001000.000001000.00000

Eigenvalues --- 1000.000001000.000001000.000001000.000001000.00000

Eigenvalues --- 1000.000001000.000001000.000001000.000001000.00000

Eigenvalues --- 1000.000001000.000001000.000001000.000001000.00000

Eigenvalues --- 1000.000001000.00000

Angle between quadratic step and forces= 51.33 degrees.

Linear search not attempted -- first point.

Iteration 1 RMS(Cart)= 0.00017869 RMS(Int)= 0.00000002

Iteration 2 RMS(Cart)= 0.00000003 RMS(Int)= 0.00000001

Variable Old X -DE/DX Delta X Delta X Delta X New X

(Linear) (Quad) (Total)

R1 2.64297 0.00001 0.00000 0.00005 0.00005 2.64302

R2 2.63409 0.00003 0.00000 0.00009 0.00009 2.63418

R3 2.71020 -0.00001 0.00000 -0.00003 -0.00003 2.71016

R4 2.63930 0.00002 0.00000 0.00005 0.00005 2.63934

R5 2.56816 -0.00005 0.00000 -0.00010 -0.00010 2.56806

R6 2.58935 -0.00001 0.00000 -0.00002 -0.00002 2.58932

R7 2.02347 0.00000 0.00000 -0.00001 -0.00001 2.02346

R8 2.65964 -0.00001 0.00000 -0.00002 -0.00002 2.65962

R9 2.02608 0.00000 0.00000 0.00000 0.00000 2.02608

R10 2.59002 0.00000 0.00000 -0.00002 -0.00002 2.59001

R11 2.02446 0.00000 0.00000 0.00001 0.00001 2.02447

R12 2.02610 0.00000 0.00000 -0.00001 -0.00001 2.02610

R13 2.01281 0.00000 0.00000 0.00001 0.00001 2.01282

R14 2.45575 -0.00001 0.00000 0.00001 0.00001 2.45576

R15 2.62656 -0.00001 0.00000 -0.00008 -0.00008 2.62648

R16 2.74163 -0.00001 0.00000 -0.00003 -0.00003 2.74160

R17 2.04704 0.00000 0.00000 0.00000 0.00000 2.04704

R18 2.03693 0.00000 0.00000 0.00001 0.00001 2.03695

R19 2.04702 0.00001 0.00000 0.00002 0.00002 2.04704

A1 2.10157 -0.00003 0.00000 -0.00012 -0.00012 2.10144

A2 1.82348 0.00001 0.00000 0.00001 0.00001 1.82348

A3 2.35814 0.00002 0.00000 0.00012 0.00012 2.35826

A4 2.11485 0.00002 0.00000 0.00009 0.00009 2.11494

A5 1.86911 -0.00001 0.00000 -0.00003 -0.00003 1.86908

A6 2.29923 -0.00001 0.00000 -0.00006 -0.00006 2.29916

A7 2.05094 0.00000 0.00000 -0.00002 -0.00002 2.05091

A8 2.11797 0.00000 0.00000 -0.00001 -0.00001 2.11797

A9 2.11428 0.00000 0.00000 0.00003 0.00003 2.11430

A10 2.12479 0.00000 0.00000 -0.00003 -0.00003 2.12476

A11 2.08266 0.00000 0.00000 0.00003 0.00003 2.08269

A12 2.07574 0.00000 0.00000 -0.00001 -0.00001 2.07573

A13 2.10583 0.00001 0.00000 0.00006 0.00006 2.10589

A14 2.07965 -0.00001 0.00000 -0.00002 -0.00002 2.07963

A15 2.09771 -0.00001 0.00000 -0.00004 -0.00004 2.09767

A16 2.06840 0.00000 0.00000 0.00002 0.00002 2.06843

A17 2.10473 0.00000 0.00000 0.00001 0.00001 2.10473

A18 2.11005 0.00000 0.00000 -0.00003 -0.00003 2.11002

A19 2.23120 0.00001 0.00000 0.00005 0.00005 2.23125

A20 1.94122 -0.00002 0.00000 -0.00004 -0.00004 1.94118

A21 2.11077 0.00001 0.00000 -0.00001 -0.00001 2.11075

A22 1.92868 0.00001 0.00000 0.00004 0.00004 1.92872

A23 2.25824 -0.00004 0.00000 -0.00022 -0.00022 2.25803

A24 2.09626 0.00003 0.00000 0.00017 0.00017 2.09644

A25 1.86230 0.00001 0.00000 0.00001 0.00001 1.86231

A26 1.93411 -0.00001 0.00000 -0.00008 -0.00008 1.93402

A27 1.86847 0.00000 0.00000 0.00001 0.00001 1.86848

A28 1.93396 0.00001 0.00000 0.00006 0.00006 1.93402

A29 1.90949 0.00000 0.00000 -0.00002 -0.00002 1.90947

A30 1.90783 0.00000 0.00000 0.00003 0.00003 1.90786

A31 1.90946 0.00000 0.00000 0.00000 0.00000 1.90947

D1 0.00004 0.00000 0.00000 -0.00004 -0.00004 0.00000

D2 -3.14152 0.00000 0.00000 -0.00008 -0.00008 3.14159

D3 -3.14159 0.00000 0.00000 -0.00001 -0.00001 -3.14159

D4 0.00005 0.00000 0.00000 -0.00005 -0.00005 0.00000

D5 -0.00030 0.00001 0.00000 0.00030 0.00030 0.00000

D6 -3.14158 0.00000 0.00000 -0.00001 -0.00001 3.14159

D7 3.14133 0.00001 0.00000 0.00026 0.00026 3.14159

D8 0.00005 0.00000 0.00000 -0.00005 -0.00005 0.00000

D9 -3.14155 0.00000 0.00000 -0.00005 -0.00005 3.14159

D10 -0.00010 0.00001 0.00000 0.00010 0.00010 0.00000

D11 0.00001 0.00000 0.00000 -0.00001 -0.00001 0.00000

D12 3.14146 0.00001 0.00000 0.00013 0.00013 -3.14159

D13 0.00020 -0.00001 0.00000 -0.00020 -0.00020 0.00000

D14 3.14154 0.00000 0.00000 0.00005 0.00005 3.14159

D15 -3.14145 0.00000 0.00000 -0.00014 -0.00014 -3.14159

D16 -0.00011 0.00001 0.00000 0.00011 0.00011 0.00000

D17 0.00002 0.00000 0.00000 -0.00002 -0.00002 0.00000

D18 3.14138 0.00000 0.00000 0.00021 0.00021 -3.14159

D19 -3.14153 0.00000 0.00000 -0.00006 -0.00006 3.14159

D20 -0.00016 0.00000 0.00000 0.00016 0.00016 0.00000

D21 -0.00016 0.00001 0.00000 0.00016 0.00016 0.00000

D22 3.14140 0.00001 0.00000 0.00019 0.00019 3.14159

D23 -3.14150 0.00000 0.00000 -0.00009 -0.00009 3.14159

D24 0.00006 0.00000 0.00000 -0.00006 -0.00006 0.00000

D25 -0.00011 0.00000 0.00000 0.00011 0.00011 0.00000

D26 -3.14152 0.00000 0.00000 -0.00007 -0.00007 3.14159

D27 3.14151 0.00000 0.00000 0.00008 0.00008 3.14159

D28 0.00010 0.00000 0.00000 -0.00010 -0.00010 0.00000

D29 0.00034 -0.00001 0.00000 -0.00034 -0.00034 0.00000

D30 -3.14157 0.00000 0.00000 -0.00002 -0.00002 3.14159

D31 -3.14144 -0.00001 0.00000 -0.00015 -0.00015 -3.14159

D32 -0.00016 0.00001 0.00000 0.00016 0.00016 0.00000

D33 0.00011 -0.00001 0.00000 -0.00011 -0.00011 0.00000

D34 3.14157 0.00000 0.00000 0.00003 0.00003 3.14159

D35 -0.00008 0.00000 0.00000 0.00008 0.00008 0.00000

D36 -3.14147 0.00000 0.00000 -0.00012 -0.00012 3.14159

D37 -1.06039 0.00000 0.00000 -0.00017 -0.00017 -1.06056

D38 -3.14148 0.00000 0.00000 -0.00011 -0.00011 3.14159

D39 1.06071 0.00000 0.00000 -0.00015 -0.00015 1.06056

D40 2.08096 0.00000 0.00000 0.00007 0.00007 2.08103

D41 -0.00013 0.00000 0.00000 0.00013 0.00013 0.00000

D42 -2.08112 0.00000 0.00000 0.00009 0.00009 -2.08103

Item Value Threshold Converged?

Maximum Force 0.000047 0.000450 YES

RMS Force 0.000011 0.000300 YES

Maximum Displacement 0.000644 0.001800 YES

RMS Displacement 0.000179 0.001200 YES

Predicted change in Energy=-3.257444D-08

Optimization completed.

-- Stationary point found.

**c) 1-naphthyl isocyanate**

-------------------

- Thermochemistry -

-------------------

Temperature 298.150 Kelvin. Pressure 1.00000 Atm.

Atom 1 has atomic number 6 and mass 12.00000

Atom 2 has atomic number 6 and mass 12.00000

Atom 3 has atomic number 6 and mass 12.00000

Atom 4 has atomic number 6 and mass 12.00000

Atom 5 has atomic number 6 and mass 12.00000

Atom 6 has atomic number 6 and mass 12.00000

Atom 7 has atomic number 6 and mass 12.00000

Atom 8 has atomic number 6 and mass 12.00000

Atom 9 has atomic number 6 and mass 12.00000

Atom 10 has atomic number 6 and mass 12.00000

Atom 11 has atomic number 6 and mass 12.00000

Atom 12 has atomic number 7 and mass 14.00307

Atom 13 has atomic number 8 and mass 15.99491

Atom 14 has atomic number 1 and mass 1.00783

Atom 15 has atomic number 1 and mass 1.00783

Atom 16 has atomic number 1 and mass 1.00783

Atom 17 has atomic number 1 and mass 1.00783

Atom 18 has atomic number 1 and mass 1.00783

Atom 19 has atomic number 1 and mass 1.00783

Atom 20 has atomic number 1 and mass 1.00783

Molecular mass: 169.05276 amu.

Principal axes and moments of inertia in atomic units:

1 2 3

EIGENVALUES -- 1234.229333050.919114285.14844

X -0.50195 0.86490 0.00000

Y 0.86490 0.50195 0.00000

Z 0.00000 0.00000 1.00000

This molecule is an asymmetric top.

Rotational symmetry number 1.

Warning -- assumption of classical behavior for rotation

may cause significant error

Rotational temperatures (Kelvin) 0.07018 0.02839 0.02021

Rotational constants (GHZ): 1.46224 0.59154 0.42116

Zero-point vibrational energy 399331.8 (Joules/Mol)

95.44260 (Kcal/Mol)

Warning -- explicit consideration of 15 degrees of freedom as

vibrations may cause significant error

Vibrational temperatures: 93.75 105.33 229.51 261.74 384.55

(Kelvin) 406.08 625.00 629.77 684.50 704.27

770.91 791.22 805.94 857.96 886.35

976.95 1027.59 1094.49 1167.05 1187.94

1212.90 1253.73 1311.44 1359.21 1436.61

1469.33 1486.05 1489.76 1507.16 1587.45

1716.19 1739.73 1754.61 1801.20 1819.28

1887.99 1969.59 1989.63 2065.63 2145.67

2164.05 2200.70 2229.01 2301.46 2345.26

2378.93 3429.25 4588.11 4596.60 4605.60

4615.57 4625.96 4636.05 4646.44

Zero-point correction= 0.152097 (Hartree/Particle)

Thermal correction to Energy= 0.161571

Thermal correction to Enthalpy= 0.162515

Thermal correction to Gibbs Free Energy= 0.116405

Sum of electronic and zero-point Energies= -550.171205

Sum of electronic and thermal Energies= -550.161731

Sum of electronic and thermal Enthalpies= -550.160787

Sum of electronic and thermal Free Energies= -550.206898

E (Thermal) CV S

KCal/Mol Cal/Mol-Kelvin Cal/Mol-Kelvin

Total 101.387 37.458 97.047

Electronic 0.000 0.000 0.000

Translational 0.889 2.981 41.283

Rotational 0.889 2.981 31.157

Vibrational 99.610 31.496 24.607

Vibration 1 0.597 1.971 4.294

Vibration 2 0.599 1.967 4.065

Vibration 3 0.621 1.892 2.556

Vibration 4 0.630 1.864 2.309

Vibration 5 0.672 1.733 1.614

Vibration 6 0.681 1.707 1.520

Vibration 7 0.795 1.395 0.844

Vibration 8 0.798 1.388 0.834

Vibration 9 0.832 1.304 0.722

Vibration 10 0.845 1.273 0.685

Vibration 11 0.891 1.171 0.574

Vibration 12 0.905 1.140 0.544

Vibration 13 0.916 1.118 0.524

Vibration 14 0.954 1.040 0.456

Vibration 15 0.976 0.998 0.423

Q Log10(Q) Ln(Q)

Total Bot 0.286783D-53 -53.542447 -123.286040

Total V=0 0.261448D+17 16.417385 37.802426

Vib (Bot) 0.230846D-67 -67.636678 -155.739206

Vib (Bot) 1 0.316711D+01 0.500663 1.152820

Vib (Bot) 2 0.281604D+01 0.449638 1.035331

Vib (Bot) 3 0.126757D+01 0.102972 0.237101

Vib (Bot) 4 0.110332D+01 0.042703 0.098327

Vib (Bot) 5 0.724083D+00 -0.140211 -0.322849

Vib (Bot) 6 0.680401D+00 -0.167235 -0.385073

Vib (Bot) 7 0.399725D+00 -0.398239 -0.916979

Vib (Bot) 8 0.395660D+00 -0.402678 -0.927201

Vib (Bot) 9 0.352823D+00 -0.452443 -1.041789

Vib (Bot) 10 0.338882D+00 -0.469952 -1.082104

Vib (Bot) 11 0.296862D+00 -0.527445 -1.214487

Vib (Bot) 12 0.285392D+00 -0.544558 -1.253891

Vib (Bot) 13 0.277422D+00 -0.556859 -1.282216

Vib (Bot) 14 0.251352D+00 -0.599717 -1.380900

Vib (Bot) 15 0.238378D+00 -0.622734 -1.433898

Vib (V=0) 0.210452D+03 2.323154 5.349259

Vib (V=0) 1 0.370634D+01 0.568945 1.310044

Vib (V=0) 2 0.336008D+01 0.526350 1.211965

Vib (V=0) 3 0.186262D+01 0.270124 0.621984

Vib (V=0) 4 0.171133D+01 0.233334 0.537271

Vib (V=0) 5 0.137994D+01 0.139861 0.322041

Vib (V=0) 6 0.134436D+01 0.128516 0.295919

Vib (V=0) 7 0.114014D+01 0.056958 0.131152

Vib (V=0) 8 0.113761D+01 0.055993 0.128930

Vib (V=0) 9 0.111195D+01 0.046086 0.106116

Vib (V=0) 10 0.110402D+01 0.042977 0.098959

Vib (V=0) 11 0.108149D+01 0.034021 0.078337

Vib (V=0) 12 0.107572D+01 0.031698 0.072986

Vib (V=0) 13 0.107181D+01 0.030116 0.069346

Vib (V=0) 14 0.105962D+01 0.025151 0.057913

Vib (V=0) 15 0.105392D+01 0.022806 0.052514

Electronic 0.100000D+01 0.000000 0.000000

Translational 0.863950D+08 7.936489 18.274440

Rotational 0.143795D+07 6.157743 14.178726

***** Axes restored to original set *****

-------------------------------------------------------------------

Center Atomic Forces (Hartrees/Bohr)

Number Number X Y Z

-------------------------------------------------------------------

1 6 0.000002375 0.000002561 0.000000000

2 6 0.000004637 0.000004063 0.000000000

3 6 -0.000000528 0.000006133 0.000000000

4 6 0.000000896 0.000003942 0.000000000

5 6 -0.000004578 0.000007002 0.000000000

6 6 0.000003273 -0.000000991 0.000000000

7 6 -0.000010791 0.000010251 0.000000000

8 6 -0.000001108 -0.000022727 0.000000000

9 6 -0.000020487 -0.000001129 0.000000000

10 6 0.000014235 -0.000004041 0.000000000

11 6 -0.000002986 0.000000447 0.000000000

12 7 0.000020383 -0.000001738 0.000000000

13 8 0.000000379 -0.000011635 0.000000000

14 1 0.000006227 -0.000001448 0.000000000

15 1 0.000004702 0.000004584 0.000000000

16 1 0.000001350 0.000008408 0.000000000

17 1 -0.000002688 0.000005291 0.000000000

18 1 -0.000003853 0.000000663 0.000000000

19 1 -0.000009130 -0.000003559 0.000000000

20 1 -0.000002309 -0.000006077 0.000000000

-------------------------------------------------------------------

Cartesian Forces: Max 0.000022727 RMS 0.000006421

GradGradGradGradGradGradGradGradGradGradGradGradGradGradGradGradGradGrad

Berny optimization.

Internal Forces: Max 0.000022412 RMS 0.000003816

Search for a local minimum.

Step number 1 out of a maximum of 2

All quantities printed in internal units (Hartrees-Bohrs-Radians)

Second derivative matrix not updated -- analytic derivatives used.

Eigenvalues --- 0.00357 0.00567 0.01468 0.01540 0.01747

Eigenvalues --- 0.01791 0.02004 0.02169 0.02273 0.02414

Eigenvalues --- 0.02445 0.02568 0.02579 0.02866 0.02995

Eigenvalues --- 0.03083 0.03306 0.03566 0.11515 0.11817

Eigenvalues --- 0.12244 0.12743 0.13114 0.13225 0.13266

Eigenvalues --- 0.15747 0.18991 0.19799 0.20186 0.20199

Eigenvalues --- 0.21016 0.21210 0.22311 0.27098 0.28527

Eigenvalues --- 0.34062 0.36045 0.36219 0.36330 0.36405

Eigenvalues --- 0.36548 0.36649 0.37058 0.38716 0.39319

Eigenvalues --- 0.40077 0.43304 0.43834 0.48643 0.48862

Eigenvalues --- 0.50263 0.51203 0.89069 1.036291000.00000

Eigenvalues --- 1000.000001000.000001000.000001000.000001000.00000

Eigenvalues --- 1000.000001000.000001000.000001000.000001000.00000

Eigenvalues --- 1000.000001000.000001000.000001000.000001000.00000

Eigenvalues --- 1000.000001000.000001000.000001000.000001000.00000

Eigenvalues --- 1000.000001000.000001000.000001000.000001000.00000

Eigenvalues --- 1000.000001000.000001000.000001000.000001000.00000

Eigenvalues --- 1000.000001000.000001000.000001000.000001000.00000

Eigenvalues --- 1000.000001000.000001000.000001000.000001000.00000

Eigenvalues --- 1000.000001000.000001000.000001000.000001000.00000

Angle between quadratic step and forces= 59.01 degrees.

Linear search not attempted -- first point.

Iteration 1 RMS(Cart)= 0.00010778 RMS(Int)= 0.00000001

Iteration 2 RMS(Cart)= 0.00000001 RMS(Int)= 0.00000000

Variable Old X -DE/DX Delta X Delta X Delta X New X

(Linear) (Quad) (Total)

R1 2.60089 0.00000 0.00000 0.00000 0.00000 2.60089

R2 2.68191 0.00000 0.00000 0.00000 0.00000 2.68191

R3 2.04544 0.00000 0.00000 0.00000 0.00000 2.04544

R4 2.67894 0.00000 0.00000 0.00001 0.00001 2.67894

R5 2.04775 0.00000 0.00000 0.00000 0.00000 2.04775

R6 2.70873 0.00000 0.00000 0.00001 0.00001 2.70873

R7 2.70398 0.00000 0.00000 0.00001 0.00001 2.70399

R8 2.60003 0.00000 0.00000 0.00000 0.00000 2.60003

R9 2.04789 0.00000 0.00000 0.00000 0.00000 2.04790

R10 2.68902 0.00001 0.00000 0.00002 0.00002 2.68904

R11 2.04962 0.00000 0.00000 0.00000 0.00000 2.04962

R12 2.68622 0.00001 0.00000 0.00001 0.00001 2.68623

R13 2.61521 0.00002 0.00000 0.00005 0.00005 2.61526

R14 2.62923 0.00001 0.00000 -0.00001 -0.00001 2.62922

R15 2.66836 0.00000 0.00000 0.00000 0.00000 2.66837

R16 2.04658 0.00000 0.00000 0.00000 0.00000 2.04658

R17 2.60038 -0.00002 0.00000 -0.00004 -0.00004 2.60034

R18 2.04731 0.00000 0.00000 0.00000 0.00000 2.04731

R19 2.04876 0.00000 0.00000 0.00000 0.00000 2.04876

R20 2.27280 -0.00001 0.00000 -0.00004 -0.00004 2.27277

R21 2.25760 0.00000 0.00000 0.00000 0.00000 2.25761

A1 2.10542 0.00000 0.00000 0.00000 0.00000 2.10542

A2 2.11260 0.00000 0.00000 0.00001 0.00001 2.11261

A3 2.06516 0.00000 0.00000 -0.00001 -0.00001 2.06515

A4 2.10129 0.00000 0.00000 0.00000 0.00000 2.10129

A5 2.09578 0.00000 0.00000 0.00001 0.00001 2.09579

A6 2.08612 0.00000 0.00000 -0.00001 -0.00001 2.08611

A7 2.08054 0.00000 0.00000 0.00000 0.00000 2.08055

A8 2.12826 0.00000 0.00000 0.00001 0.00001 2.12827

A9 2.07438 0.00000 0.00000 -0.00001 -0.00001 2.07436

A10 2.09911 0.00000 0.00000 0.00000 0.00000 2.09911

A11 2.08663 0.00000 0.00000 -0.00001 -0.00001 2.08662

A12 2.09744 0.00000 0.00000 0.00001 0.00001 2.09745

A13 2.10967 0.00000 0.00000 0.00000 0.00000 2.10968

A14 2.10556 0.00000 0.00000 0.00000 0.00000 2.10556

A15 2.06795 0.00000 0.00000 0.00000 0.00000 2.06795

A16 2.07033 0.00000 0.00000 -0.00001 -0.00001 2.07032

A17 2.08264 0.00000 0.00000 0.00001 0.00001 2.08265

A18 2.13021 0.00000 0.00000 0.00000 0.00000 2.13021

A19 2.09531 0.00000 0.00000 -0.00001 -0.00001 2.09530

A20 2.06038 0.00000 0.00000 0.00001 0.00001 2.06039

A21 2.12750 0.00000 0.00000 0.00000 0.00000 2.12750

A22 2.10612 0.00000 0.00000 0.00000 0.00000 2.10612

A23 2.08676 0.00000 0.00000 -0.00001 -0.00001 2.08675

A24 2.09030 0.00000 0.00000 0.00001 0.00001 2.09031

A25 2.10628 0.00000 0.00000 0.00000 0.00000 2.10627

A26 2.07623 0.00000 0.00000 0.00002 0.00002 2.07624

A27 2.10068 0.00000 0.00000 -0.00002 -0.00002 2.10067

A28 2.10164 0.00000 0.00000 0.00001 0.00001 2.10166

A29 2.07510 0.00000 0.00000 0.00001 0.00001 2.07510

A30 2.10644 0.00000 0.00000 -0.00002 -0.00002 2.10642

A31 2.56651 0.00001 0.00000 0.00030 0.00030 2.56680

A32 3.24931 0.00000 0.00000 -0.00006 -0.00006 3.24925

A33 3.14159 0.00000 0.00000 0.00000 0.00000 3.14159

D1 0.00000 0.00000 0.00000 0.00000 0.00000 0.00000

D2 3.14159 0.00000 0.00000 0.00000 0.00000 3.14159

D3 3.14159 0.00000 0.00000 0.00000 0.00000 3.14159

D4 0.00000 0.00000 0.00000 0.00000 0.00000 0.00000

D5 0.00000 0.00000 0.00000 0.00000 0.00000 0.00000

D6 3.14159 0.00000 0.00000 0.00000 0.00000 3.14159

D7 3.14159 0.00000 0.00000 0.00000 0.00000 3.14159

D8 0.00000 0.00000 0.00000 0.00000 0.00000 0.00000

D9 0.00000 0.00000 0.00000 0.00000 0.00000 0.00000

D10 3.14159 0.00000 0.00000 0.00000 0.00000 3.14159

D11 3.14159 0.00000 0.00000 0.00000 0.00000 3.14159

D12 0.00000 0.00000 0.00000 0.00000 0.00000 0.00000

D13 0.00000 0.00000 0.00000 0.00000 0.00000 0.00000

D14 3.14159 0.00000 0.00000 0.00000 0.00000 3.14159

D15 3.14159 0.00000 0.00000 0.00000 0.00000 3.14159

D16 0.00000 0.00000 0.00000 0.00000 0.00000 0.00000

D17 3.14159 0.00000 0.00000 0.00000 0.00000 3.14159

D18 0.00000 0.00000 0.00000 0.00000 0.00000 0.00000

D19 0.00000 0.00000 0.00000 0.00000 0.00000 0.00000

D20 3.14159 0.00000 0.00000 0.00000 0.00000 3.14159

D21 0.00000 0.00000 0.00000 0.00000 0.00000 0.00000

D22 3.14159 0.00000 0.00000 0.00000 0.00000 3.14159

D23 3.14159 0.00000 0.00000 0.00000 0.00000 3.14159

D24 0.00000 0.00000 0.00000 0.00000 0.00000 0.00000

D25 0.00000 0.00000 0.00000 0.00000 0.00000 0.00000

D26 3.14159 0.00000 0.00000 0.00000 0.00000 3.14159

D27 3.14159 0.00000 0.00000 0.00000 0.00000 3.14159

D28 0.00000 0.00000 0.00000 0.00000 0.00000 0.00000

D29 0.00000 0.00000 0.00000 0.00000 0.00000 0.00000

D30 3.14159 0.00000 0.00000 0.00000 0.00000 3.14159

D31 3.14159 0.00000 0.00000 0.00000 0.00000 3.14159

D32 0.00000 0.00000 0.00000 0.00000 0.00000 0.00000

D33 0.00000 0.00000 0.00000 0.00000 0.00000 0.00000

D34 3.14159 0.00000 0.00000 0.00000 0.00000 3.14159

D35 3.14159 0.00000 0.00000 0.00000 0.00000 3.14159

D36 0.00000 0.00000 0.00000 0.00000 0.00000 0.00000

D37 3.14159 0.00000 0.00000 0.00000 0.00000 3.14159

D38 0.00000 0.00000 0.00000 0.00000 0.00000 0.00000

D39 0.00000 0.00000 0.00000 0.00000 0.00000 0.00000

D40 3.14159 0.00000 0.00000 0.00000 0.00000 3.14159

D41 3.14159 0.00000 0.00000 0.00000 0.00000 3.14159

D42 0.00000 0.00000 0.00000 0.00000 0.00000 0.00000

D43 0.00000 0.00000 0.00000 0.00000 0.00000 0.00000

D44 3.14159 0.00000 0.00000 0.00000 0.00000 3.14159

D45 3.14159 0.00000 0.00000 0.00000 0.00000 3.14159

D46 0.00000 0.00000 0.00000 0.00000 0.00000 0.00000

Item Value Threshold Converged?

Maximum Force 0.000022 0.000450 YES

RMS Force 0.000004 0.000300 YES

Maximum Displacement 0.000778 0.001800 YES

RMS Displacement 0.000108 0.001200 YES

Predicted change in Energy=-3.110932D-09

Optimization completed.

-- Stationary point found.
